# Introducing Novel Methods to Identify Fraudulent Responses (Sampling With Sisyphus): Web-Based LGBTQ2S+ Mixed-Methods Study

**DOI:** 10.2196/63252

**Published:** 2025-03-17

**Authors:** Kinnon Ross MacKinnon, Naail Khan, Katherine M Newman, Wren Ariel Gould, Gin Marshall, Travis Salway, Annie Pullen Sansfaçon, Hannah Kia, June SH Lam

**Affiliations:** 1 School of Social Work York University Toronto, ON Canada; 2 Dalla Lana School of Public Health University of Toronto Toronto, ON Canada; 3 Department of Psychology York University Toronto, ON Canada; 4 Faculty of Health Sciences Simon Fraser University Vancouver, BC Canada; 5 School of Social work University of Montreal Montreal, QC Canada; 6 University of Stellenbosch Stellenbosch South Africa; 7 School of Social Work The University of British Columbia Vancouver, BC Canada; 8 Temerty Faculty of Medicine University of Toronto Toronto, ON Canada; 9 Gender Identity Clinic Centre for Addiction and Mental Health Toronto, ON Canada

**Keywords:** sampling, bots, transgender, nonbinary, detransition, lesbian, gay, bisexual, and transgender, mobile phone

## Abstract

**Background:**

The myth of Sisyphus teaches about resilience in the face of life challenges. Detransition after an initial gender transition is an emerging experience that requires sensitive and community-driven research. However, there are significant complexities and costs that researchers must confront to collect reliable data to better understand this phenomenon, including the lack of a uniform definition and challenges with recruitment.

**Objective:**

This paper presents the sampling and recruitment methods of a new study on detransition-related phenomena among lesbian, gay, bisexual, transgender, queer, and 2-spirit (LGBTQ2S+) populations. It introduces a novel protocol for identifying and removing bot, scam, and ineligible responses from survey datasets and presents preliminary descriptive sociodemographic results of the sample. This analysis does not present gender-affirming health care outcomes.

**Methods:**

To attract a large and heterogeneous sample, 3 different study flyers were created in English, French, and Spanish. Between December 1, 2023, and May 1, 2024, these flyers were distributed to >615 sexual and gender minority organizations and gender care providers in the United States and Canada, and paid advertisements totaling >CAD $7400 (US $5551) were promoted on 5 different social media platforms. Although many social media promotions were rejected or removed, the advertisements reached >7.7 million accounts. Study website visitors were directed from 35 different traffic sources, with the top 5 being Facebook (3,577,520/7,777,218, 46%), direct link (2,255,393/7,777,218, 29%), Reddit (1,011,038/7,777,218, 13%), Instagram (466,633/7,777,218, 6%), and X (formerly known as Twitter; 233,317/7,777,218, 3%). A systematic protocol was developed to identify scam, nonsense, and ineligible responses and to conduct web-based Zoom video platform screening with select participants.

**Results:**

Of the 1377 completed survey responses, 957 (69.5%) were deemed eligible and included in the analytic dataset after applying the exclusion protocol and conducting 113 virtual screenings. The mean age of the sample was 25.87 (SD 7.77; median 24, IQR 21-29 years). A majority of the participants were White (Canadian, American, or of European descent; 748/950, 78.7%), living in the United States (704/957, 73.6%), and assigned female at birth (754/953, 79.1%). Many participants reported having a sexual minority identity, with more than half the sample (543/955, 56.8%) indicating plurisexual orientations, such as bisexual or pansexual identities. A minority of participants (108/955, 11.3%) identified as straight or heterosexual. When asked about their gender-diverse identities after stopping or reversing gender transition, 33.2% (318/957) reported being nonbinary, 43.2% (413/957) transgender, and 40.5% (388/957) identified as detransitioned.

**Conclusions:**

Despite challenges encountered during the study promotion and data collection phases, a heterogeneous sample of >950 eligible participants was obtained, presenting opportunities for future analyses to better understand these LGBTQ2S+ experiences. This study is among the first to introduce an innovative strategy to sample a hard-to-reach and equity-deserving group, and to present an approach to remove fraudulent responses.

## Introduction

### Background

In the past several years, the topic of detransition has garnered significant attention in the mainstream media and academic literature as an emerging sociomedical phenomenon in need of further research and care provision [1-3]. Detransition is an umbrella term inclusive of stopping, shifting, or reversing the social, legal, and medical interventions undertaken during an initial gender transition [4-6]. Depending on the social, legal, and medical steps taken for an initial gender transition, detransition may include shifting sexual and gender minority (SGM) identities and gender expression, social detransition such as reverting to using pronouns and a gender expression aligned with the assigned-at-birth gender, legal change of name or gender marker, and the discontinuation or reversal of gender-related medical treatments [4,5,7]. Transgender and gender-diverse (TGD) people may stop taking gender-related hormonal treatments for reasons unrelated to identity shifts, and it is important to recognize that detransition is distinct from—but sometimes overlaps with—the temporary or permanent discontinuation of gender-related medical care. Depending on definitions and measurement decisions, studies conducted in the United States, Canada, the United Kingdom, Australia, and the Netherlands estimate that between 1% and 13.1% of TGD people experience detransition at some point in their lives [4,8-10]. However, due to study designs, it can be difficult to know the extent to which stopping treatments or detransition is temporary or permanent.

Researchers, gender-affirming care guidelines, and TGD scholars themselves have highlighted a need for qualitative and quantitative research to understand long-term outcomes after gender transition, including detransition and gender identity evolution [10-14]. However, several challenges can hamper efforts to produce this knowledge. Topping the list includes the stigmatization and politicization of detransition, concerns that publishing negative or unexpected results about gender transition are being weaponized against TGD populations and access to gender care, and a lack of scholarly agreement about concepts—all of which contribute to limitations in studying this issue [7].

There are also opportunities to overcome these barriers by using comprehensively designed, SGM (eg, lesbian, gay, bisexual, transgender, queer, and 2-spirit [LGBTQ2S+]) community–led research studies. Nonprobability web-based sampling and recruitment offers specific strengths in researching gender minority populations [15]. This approach to recruitment is recommended to periodically explore health and social issues affecting LGBTQ2S+ communities [16], and it has been shown to be effective alongside in-person recruitment [17]. However, there are important considerations and challenges in conducting web-based research generally; for example, web-based research has always presented issues with fraudulent or “impostor” responses to surveys, but this activity has surged since the COVID-19 pandemic [18]. Many research projects have been affected by bots and impostor responders, often motivated by financial incentive [19,20].

The integrity of web-based research faces threats on several fronts; for example, identifying and removing bot, scam, or ineligible responses from datasets is an emerging priority [21,22]. TGD and detransition research, in particular, has been subject to scam responses and sabotage efforts [23-25]. Haverkamp et al [23] identified that 50 out of 349 responses to a web-based survey designed for TGD students included slurs or hate speech. One detransition study received invalid responses to skew results [24]. While web-based survey platforms often include automatic bot detection methods such as CAPTCHA (Completely Automated Public Turing Test to Tell Computers and Humans Apart) [21] and the identification of nonunique IP addresses, these alone are not effective at identifying fraudulent or malicious responses [26]. Additional techniques used to identify and deter scam responses include collecting IP addresses or geolocation data, conducting video screenings, and incorporating scam detection questions that legitimate participants would not answer affirmatively; for example, the study by Hays et al [27] included asking participants about being diagnosed with fictitious conditions. Participants who affirmed such diagnoses were deemed ineligible. Other studies have set exclusion criteria based on mining survey responses for specific nonsense or incoherent responses [25]. A technique that has been used in studies of detransition involves personal video interviews, which remove anonymity and require participants to verify demographic details such as age and location to ensure consistency with their survey responses [24]. Requiring participants to appear on camera has also been identified as a deterrent to scam responses [24,25].

### Objectives

Following from these considerations, we present a novel sampling and recruitment methodology of a study designed to generate knowledge about detransition. Its aims are four-fold: (1) to present survey design, sampling, and recruitment decisions; (2) to illustrate comprehensive study design guidance for future researchers who are studying detransition-related phenomena; (3) to highlight a protocol for identifying and excluding scam, nonsense, and ineligible survey responses; and (4) to present sociodemographic study results that demonstrate that these efforts obtained a heterogeneous SGM sample.

The Detransition Analysis, Representation, and Exploration (DARE) study was launched in December 2023 to understand sociodemographic characteristics, LGBTQ2S+ identities, life histories, minority stressors, and gender care encounters of individuals with experience of stopping, shifting, or reversing an initial gender transition (eg, detransition). This project was designed to overcome challenges in reaching people with these experiences. The study design, sampling, and study promotion decisions were all made purposefully to include individuals connected with—or disconnected from—gender care providers, organizations serving TGD and LGBTQ2S+ populations, and web-based networks for detransitioned (detrans) people. These decisions aimed to mitigate sampling bias and limitations from past research and to include hard-to-reach disparate TGD and detrans communities with the goal of building theoretical, empirical, and practice-oriented knowledge.

### Exploratory Theories of Detransition Developed From Community and Clinical Samples

Diverse theories proposed to understand pathways to detransition have been developed via community and clinical samples, and they comprise internal (eg, internal change in SGM identity and medical complications), system-level (eg, health care availability and access), and external (discrimination, stigma, and lack of support for TGD identities) factors. Taken together, two primary experiences seem to occur with detransition: (1) a shift in one’s internal self-conceptualization of sex, gender, and/or sexual orientation identity *after* an initial gender transition and (2) discontinuation or reversal of *prior* gender-affirming medical, legal, and social interventions [4,24,28-32].

However, existing theories should be considered preliminary, given the issues of study design and methodological limitations; for instance, disparate conceptualizations of detransition are applied in the literature, and there are issues of sampling and selection bias and potentially unreliable data collection instruments. Notable examples include analyses of medical case notes [32-34], community surveys administered to TGD populations [9], and surveys administered to web-based detrans communities [24,30,35].

### Sampling and Selection Bias in Clinical and Community-Derived Samples

Prior research examining detransition has been limited by sampling strategy, often due to restrictive inclusion criteria such as reidentifying with birth-assigned gender to be counted as a detransition; for instance, studying detransition with clinical data (eg, medical case notes and recruiting from health care clinics) carries several limitations. North American gender clinics rarely have the resources to conduct long-term follow-up, and some TGD people emigrate; decline to participate in research studies; or move from pediatric to adult services, which can introduce survivorship bias [7]. Case series studies carry the risk of excluding patients who discontinue treatment, detransition, and disconnect from care or avoid care providers due to feelings of shame or fear of judgment—experiences identified in past research [28,35,36]. Short data collection periods in clinical samples can also hinder estimating detransition prevalence and understanding identity evolution that—when these outcomes do occur—often happen several years after initiating a medical transition [5,37]. Community-derived samples obtained via organizations and social media can provide useful data on individuals who are no longer connected to the same care providers or the health care system and who are otherwise considered hard to reach due to systemic marginalization.

One notable example is the 2015 United States Transgender Survey (USTS)—a large cross-sectional nonprobability community-driven study [9]. Finding that detransition among TGD adults is largely driven by external pressures such as lack of support and social stigma, Turban et al [9] analyzed a subsample of the 2015 USTS dataset—17,151 TGD adults who had ever initiated a gender transition. The authors found that 2242 (13.07%) of the 17,151 participants responded positively to the question “Have you ever de-transitioned? In other words, have you ever gone back to living as your sex assigned at birth, at least for a while?” Between the cross-sectional design of the study and the wording of this question, it is difficult to know whether respondents detransitioned temporarily, permanently, or both. The survey, developed by the National Center for Transgender Equality, favored external factors and pressure-related reasons in its predetermined list of reasons for detransition. Participants were shown 10 reasons for detransition that included external factors and pressure-related reasons (eg, “pressure from a parent” and “pressure from family members”), and they were shown only 2 nonexternal reasons (ie, “I realized gender transition was not for me” and “not listed above [please specify]”). Study promotion and recruitment efforts comprehensively targeted 800 TGD-serving, LGBTQ2S+, and allied community organizations [38]. Support groups for detransitioning were likely nonexistent at the time of data collection and were thus not involved in study promotion or recruitment. Moreover, individuals with a history of gender transition who detransitioned and no longer held a TGD identity would have been excluded per the USTS inclusion criteria [38]. A majority of the respondents were TGD adults aged ≥25 years (65.5%) and transfeminine individuals assigned male at birth (AMAB; 55%).

Similarly, cross-sectional community surveys conducted through web-based detrans networks present limitations in sampling and study-related decisions that prioritize detransitioned individuals who stopped identifying as TGD or disconnected from TGD communities. The survey conducted in 2019 [39] by Vandenbussche [30] aimed to explore the care needs of the web-based detrans community via the Post Trans project [40] and other web-based groups largely serving female detransitioners on Facebook, Instagram, Twitter (subsequently rebranded as X), and Reddit (r/detrans). No TGD or LGBTQ2S+ organizations seem to have been involved in participant recruitment [30]. The survey recruited 237 participants (92% female or assigned female at birth [AFAB] individuals). Of this sample, 65% transitioned both socially and medically, while 31% transitioned only socially. Although the survey provided a question on gender and gender identity (“How do you see yourself now?” with the following response options: “woman,” “man,” “trans man,” “trans woman,” “female detransitioner,” “male detransitioner,” “non-binary,” and “other”), no results are presented to discern whether any participants in the sample affirmed a TGD identity when they took the survey—participants are referred to as either “female detransitioners” or “male detransitioners.”

Another survey of 100 people (69% female or AFAB individuals) who discontinued or reversed gender-related medical interventions recruited participants from largely web-based sources from December 2016 to April 2017 (4.5 mo) via Twitter, Tumblr, Reddit, and closed detransition groups [35]. This survey was also circulated on professional listserves via the American Psychological Association, the World Professional Association of Transgender Health, and SEXNET. The inclusion criteria required participants to have undergone medical transition (those with only social transition experiences were excluded). While participants in this survey had the option to indicate a current TGD identity, only 39% reported a TGD identity.

In comparison to the 2015 USTS instrument that prioritized external pressure–related reasons for detransition, the surveys administered by Vandenbussche [30] and Littman [35] favored internal, psychological, and physical health–related factors in their predetermined reasons for detransition. However, these surveys also included structural (eg, financial barriers) and externally driven factors, such as a lack of support and discrimination. Both surveys recruited a majority of female or AFAB individuals and favored individual-level driving factors motivating detransition. However, these studies were limited by small sample sizes and selection bias in that the recruitment and inclusion criteria focused primarily on individuals who understood themselves as *detransitioners* or as having been *detransitioned* at the time of data collection—terminology that can be divisive [41].

Some detransitioned people report experiencing rejection from TGD and LGBTQ2S+ communities and organizations [30,42], meaning that these individuals may be hard to reach for investigators whose networks are composed primarily of LGBTQ2S+ contacts. Therefore, sampling and recruitment that takes a broad approach is necessary to include individuals who may have become disconnected from SGM networks.

## Methods

### Study Population

To overcome past limitations and to understand the life experiences, minority stressors, and SGM identities of people who self-identify with experiences related to detransitioning, we conducted a binational, cross-sectional, mixed methods study in Canada and the United States. Our objective was to target a large and heterogeneous sample of 500 to 1000 participants (including detrans, TGD, and LGBTQ2S+ people). This target sample size of >500 was selected due to the anticipated heterogeneity of the group and requiring a large sample to explore and characterize detransition broadly [43]. The mixed quantitative and qualitative approach was selected to quantify different pathways (reasons for detransitioning) and to understand qualitatively the care needs specific to various pathways.

### Inclusion and Exclusion Criteria

To be eligible, participants had to be aged ≥16 years; able to fill out a survey in English, Spanish, or French; and had to report a life experience of stopping, shifting, or reversing a gender transition. Gender transition was defined as inclusive of social, legal, and medical transition, and it was not a requirement to have accessed gender-related medical treatments to be eligible. We included social-only, social+medical, and medical-only transition experiences. This decision was supported by the range of inclusion criteria from past studies [9,30,41,42,44] and aligned with our aim of building knowledge about individuals who self-identify with detransition-related experiences. As has been pointed out, some individuals may detransition socially or medically and continue to affirm a TGD identity, while others may shift from a TGD identity to a detransitioner identity without ever being able to detransition socially or medically [4,30,41]. Recruitment and study promotional materials stated that the study was open to anyone with a history of shifting gender identity; stopping or reversing a gender transition; or detransitioning for any reason, such as a loss of access to gender-affirming health care, medical complications, misdiagnosis, discrimination and lack of support, or an identity change.

To be eligible, participants also had to be living in the United States or Canada during the study period, and IP addresses and geolocation data were collected. The United States was included as a geographic region of interest, in addition to Canada, due to the US political and legislative context surrounding gender-affirming health care restrictions [45], antitransgender rhetoric, and proximity to Canada [46]. As it is likely that some TGD people may be forced to detransition due to the reduced availability of gender-affirming health care, legislative restrictions, and antitransgender rhetoric, one of the DARE study flyers was designed specifically to invite participants who were forced to stop transitioning or detransition due to the loss of gender-affirming health care.

The minimum age for participation was set at 16 years for 3 reasons. First, many Canadian research ethics boards recognize this as the minimum legal age for providing independent informed consent for research studies. Second, we aimed to examine the unique experiences of detransitioning during the teen years compared to adulthood. Third, given that the time between transition and detransition can span years, setting the minimum age at 16 years meant that we would be likely to include some individuals who began their initial transition in childhood. No maximum age limit was imposed to ensure a diverse sample with broad sociodemographic backgrounds and life experiences.

### Survey Development and Measures

#### Reflexivity and Community Engagement

The English-language survey was developed collaboratively by the full DARE study team—a multidisciplinary group of social work and public health researchers who study the health and well-being of SGM populations. The team includes a majority of TGD (spanning the transfeminine and transmasculine spectrums) and SGM individuals and includes a gender-affirming clinician. Following the recommendations by Hildebrand-Chupp [41] on language considerations for detrans research, we developed the survey using neutral language designed to be sensitive to diverse TGD and detrans populations; for example, after detransition, some individuals feel affirmed by terms and language associated with sex rather than gender identity, and past qualitative studies with detrans young people illustrate that some feel that they do not have a gender identity [28]. To be inclusive of all respondents, the survey posed the question “What term(s) best describe your current *gender?*” rather than asking about *gender identity*. Participants were also asked a series of questions about whether, after stopping or reversing transition, they understand themselves as transgender, nonbinary, cisgender, or detransitioned (the response options were “yes,” “no,” and “unsure”). These questions were not mutually exclusive.

We used community-engaged research strategies. The survey was reviewed for language accuracy by an English-speaking person in the United States with experience of detransition who received a gift card valued at US $150. The survey was initially pilot-tested in English by members of the DARE study team (n=9). Subsequently, external pilot testers were recruited through personal and professional networks, including TGD and non-TGD detransitioned people, and a gender care provider—including AFAB and AMAB individuals (n=4). Pilot testers were asked to comment on the appropriateness of the language, survey completion time, the flow and organization of the survey, broken links, the coherent ordering of questions, questions that were challenging to understand, and overall comments. After pilot testing, the revised English survey was translated into French and Spanish. The translated French and Spanish surveys were reviewed and pilot-tested by native speakers. External pilot testers received gift cards worth CAD $50 (US $37) or US $50, depending on their country of residence.

#### Survey Development

The comprehensive survey was programmed in Qualtrics (Qualtrics International Inc) and made available through a Qualtrics platform accessible from a dedicated web page. On the basis of pilot testing, it was anticipated that participants would take 35 to 60 minutes to complete the survey. It was programmed using skip logics that were sensitive to individualized transition experiences reported by participants (those who had engaged in both a social and a medical transition were shown the greatest number of questions; those responding “no” to initiating a medical transition were not shown questions pertaining to medical or surgical interventions). The survey included extensive sociodemographic items and integrated measures from several prior health questionnaires delivered to SGM communities [47] and individuals who had detransitioned [9,35].

To ensure that the survey was community and data driven as well as inclusive of a wide range of detransition pathways, participants were presented with a Likert scale listing 21 possible reasons for stopping or reversing their initial gender transition. The Likert scale was developed from prior empirical studies with TGD and detransitioned people [9,28-31,35,48]. The survey transparently highlighted 4 different thematic categories, prompting participants to indicate to what extent a cluster of various reasons contributed to their decision (Multimedia Appendix 1). Whereas Turban et al [9] retrospectively organized reasons for detransition into “internal” and “external” reasons, the DARE survey explicitly organized reasons a priori into 4 dimensions. Participants were shown grouped factors as follows: (1) mental health or psychological reasons (eg, “My mental health did not improve while transitioning” and “My mental health was worse while transitioning”), (2) physical reasons (eg, “Satisfied with the physical results of transition” and “My physical health was worse while transitioning”), (3) external reasons (eg, “I felt discriminated against,” “I did not have enough support in my life to continue transitioning,” “I had trouble paying for hormones or surgery,” and “Legislative bans on gender care required me to stop transitioning”), and (4) social or internal reasons (eg, “My personal definition of woman or man changed and I became more comfortable with my birth sex” and “My identity changed and I no longer felt a need for medical interventions”). Unlike prior studies, participants were asked to rate these reasons using a Likert scale (eg, “not at all,” “a little,” “somewhat,” and “a lot”) rather than categorically (“yes” or “no”) to reflect the possibility that participants may cite multidimensional pathways.

### Study Promotion and Participant Recruitment

#### Overview

To provide information about the study and direct prospective participants to the informed consent form and survey, an English-language website was created. This website featured a promotional video about the study and provided buttons linking to the English, French, and Spanish versions of the consent form and survey. The website also contained information about the study funder, the study team, project objectives, and recruitment flyers.

Study promotion materials were intentionally broad and neutral due to narratives of detransition being polarized. As the study objective was to reach heterogeneous populations, including TGD, SGM, and non-TGD detransitioned people, we aimed to be inclusive. To reach a diverse sample, 3 different tailored recruitment flyers were designed with a variety of language choices and color schemes that were anticipated to attract people with a range of experiences (Multimedia Appendix 2). One flyer used language that focused on attracting TGD people who had experienced shifts in gender identity, and another applied TGD-coded colors (pink and blue) to attract participants who were forced to detransition due to external factors. A third flyer used colors (green and blue) and language choices anticipated to attract detrans populations who no longer identify as TGD. The term *detransition* was included on all recruitment materials, the study website, and in the informed consent form. The French and Spanish versions of these 3 flyers were also advertised widely across social media platforms and distributed to organizations and care providers in the United States and Canada.

#### LGBTQ2S+ Organizations and Gender Care Providers

Direct invitation emails were sent to >615 TGD or LGBTQ2S+ organizations and gender care providers, most of them serving English-speaking or multilingual populations. Efforts were made to contact organizations, gender clinics, and gender therapists who work with TGD, LGBTQ2S+, and detransitioning people. The direct emails disproportionately focused on English speakers because French and Spanish are spoken by a minority of people in North America. Hence, 21 French-speaking organizations were contacted directly by email, 5 French-speaking organizations were contacted via LinkedIn, and the flyer was shared directly with Francophone groups for parents of TGD individuals. In addition, 9 Spanish-speaking organizations were contacted. The Spanish recruitment flyers were shared on social media by another researcher studying Spanish-speaking TGD and detrans populations.

We offered to meet with 5 LGBTQ2S+ youth groups in the Greater Toronto Area, Ontario, Canada, to engage in direct study promotion and recruitment and answer questions about the study. None of these organizations responded to the offer. One LGBTQ2S+ organization in Toronto; 1 organization serving TGD populations in British Columbia, Canada; and at least 2 gender clinics in the United States and in Canada confirmed posting physical recruitment flyers in high-traffic areas to support recruitment efforts. There may have been additional physical posters publicized by organizational contacts without our awareness.

#### Webinar

Toward the end of the data collection phase in April 2024, we privately invited select stakeholders from LGBTQ2S+ organizations, gender clinics, and TGD and detransitioned people within our networks to a webinar presenting preliminary data to aid in further study promotion. Of the 83 people who registered for the event, 38 (46%) attended. The webinar presented the objectives and methodology of the study, preliminary results, and ended with a direct request for support with participant recruitment. The webinar was not recorded or posted on the web.

#### Former LGBTQ2S+ Research Participants

Participants from prior team research studies who had consented to being contacted for future research opportunities were emailed information about the study. These were largely Canadian former participants of 2 different studies related to LGBTQ2S+ affirmation (N=1181) and detransition and identity fluidity (N=27). The majority (1014/1208, 83.9%) were Anglophone and sent English recruitment materials; the rest (194/1208, 16.1%) were Francophone and sent French recruitment materials.

#### Paid and Unpaid Social Media Advertisements

Between December 1, 2023, and May 1, 2024, flyers in all 3 study languages were widely distributed on the web via paid and unpaid social media posts and advertisements across TikTok, Instagram, Facebook, Tumblr, X, Reddit, Discord, and Grindr (Multimedia Appendices 3-5). Where possible, we also advertised in English-speaking private groups on social networking sites for TGD and detrans people, as well as parents of TGD youth. Of note, the DARE study principal investigator maintains a TikTok account with >29,500 followers, where they discuss TGD and detrans research. To enhance engagement and promote the study using audiovisuals, several TikTok videos about the study, as well as YouTube videos, were produced and shared on the web. TikTok has a powerful algorithm that is able to reach large numbers of people and is recognized as a useful platform in community-engaged TGD and gender care knowledge sharing [49]. To estimate the reach and impact of our advertisements, at the end of the data collection period, we reviewed impressions, likes, and shares across all social media platforms used. We summed analytics data from each platform.

### Excluding Scam and Ineligible Survey Responses

To enhance the reliability of the survey data, we followed guidance from prior studies [25,26,50]. Adding 2 fictitious conditions—“chekalism” and “syndomitis”—has been recommended as an effective way of identifying scam responses [27]. These 2 items were included in the survey and shown to all participants (eg, “At any point while transitioning, did you develop chekalism?” with the following response options: “yes,” “no,” and “unsure”). We further flagged potential scam responses based on nonsense, incoherent, or hateful email addresses, as advised by Pullen Sansfaçon et al [25] and Haverkamp et al [23].

To remove bot, scam, and ineligible responses from the dataset, a protocol was developed using guidelines and recommendations from previous studies. First, we removed any responses from IP addresses that had submitted multiple completed surveys [50], as well as those originating from outside the United States or Canada (based on IP address and geolocation data). Next, to remove any bot responses, we followed the guidelines suggested by Google and Qualtrics developers and excluded any responses with a reCAPTCHA score of <0.5 [26]. We then excluded respondents who indicated that they had neither socially nor medically transitioned (they were deemed ineligible based on the study’s inclusion and exclusion criteria). Participants who indicated “yes” to having either of the 2 fictitious conditions were also excluded. In addition, we left out participants who reported no discordance between their past and current gender, as well as those whose reported sex assigned at birth conflicted with the hormone they indicated taking during their initial medical transition (eg, a female or AFAB individual who reported taking estrogen or a male or AMAB individual who reported taking testosterone). Survey responses with a completion time of <12 minutes were also removed, given that pilot testing estimated a typical completion time of 35 to 60 minutes.

Participants identified as potentially scam or ineligible for the study who had consented to being contacted were invited via email to complete a screening interview over Zoom (Zoom Video Communications, Inc) to verify their survey responses. If the research team could validate their eligibility and the accuracy of their survey responses, their survey data were readded to the dataset. As part of the screening process, interview participants were required to turn on their cameras for identification at the beginning of the interview, following guidelines suggested by previous literature [18,25]. Each screening interview was audio recorded. The screening questions were designed to be easy for genuine participants to answer. Those interviewing potential scam participants were also asked to document additional indicators of scams, such as poor or unusual audio quality and sounds, inconsistencies in daylight relative to the participant’s reported time zone, and vague and brief responses [25]. The screening interview included the following six questions:

1. Can you please confirm your age?
2. Can you please confirm the country, province (or state), and city you are currently living in?
3. Can you please tell us the country, province (or state), and city you were in when you accessed the survey?
4. Can you please confirm what terms best describe your current gender?
5. For your initial transition, did you socially transition, and if so, in which year?
6. Could you please describe the main reasons you detransitioned?

Participants who could not answer these questions or provided answers inconsistent with their survey responses were excluded. During the process of screening potential scam responses, team members had participants select an interview time via the Calendly website, which allowed the interviewer to confirm the participant’s email address and time zone. This verification process was also applied to 4 gift card raffle winners and participants who were screened before the qualitative interview phase.

We removed from the analytic sample all potential scam and ineligible respondents flagged by this protocol who did not consent to being contacted by the research team, did not provide a follow-up email, or did not respond to an invitation to complete the Zoom interview. During the screening process, 2 recurring anomalies were observed: first, Calendly showed an unexpected number of sign-ups from the West African time zone; second, there were several sign-ups for screening from email addresses that we had not personally contacted. This suggests that our email invitation to the screening process had been shared with additional individuals who had never participated in the DARE survey.

### Ethics Approval

The study (protocol #3964) received ethics approval from York University (e2023-298), and all participants provided written informed consent to take the survey. This consent included providing explicit agreement to publishing anonymized data for research. Most survey questions were optional, and participants could exit the survey at any time. At any point while taking the survey, participants could access a list of mental health care resources for youth and adults. After providing informed consent, participants were given the opportunity to enter a raffle for a US $50 or CAD $50 (US $37) gift card, which was also advertised in study promotional materials.

## Results

### Overview

Between December 1, 2023, and May 1, 2024, we widely distributed 3 different study flyers in English, French, and Spanish via direct emails and social media platforms. Paid and unpaid advertising on social media served as a primary recruitment strategy, and paid advertisements totaling CAD $7494.81 (US $5551.71) were promoted on 5 different platforms that targeted >40 unique groups and networks (Table 1; Multimedia Appendices 4 and 5).

**Table 1:** Paid advertisements (December 13, 2023-April 25, 2024) per social media platform (total cost: CAD $7494.81 [US $5551.71]).

Social media platforms	Cost (CAD $^a^), n (%)
Grindr (US $ converted to CAD $)	1028.60 (13.7)
Instagram and Facebook	2808.18 (37.5)
Reddit	2166.34 (28.9)
TikTok	659.40 (8.8)
Tumblr (US $ converted to CAD $)	832.29 (11.1)
X (no paid advertising accepted)	0 (0)

^a^A currency exchange rate of CAD $1=US $1.35 is applicable.

According to data provided by each social media platform, paid and unpaid study advertisements reached >7.7 million accounts (Table 2). Although efforts were made to equally promote all study posters in 3 languages, discrepancies arose because some posts seemed to receive more positive or negative attention and engagement, which likely affected engagement levels and overall promotion and views. Analytics data from TikTok indicated that flyer 3 (“forced detransition”) received a higher number of shares between users and “likes,” which may have amplified its views and impressions (Multimedia Appendix 3). During the study promotion period, 30 different Reddit subreddits were targeted via paid advertisements totaling CAD $2166.34 (US $1604.69) and unpaid posts from our study team or other users (Table 1; Multimedia Appendix 4). Instagram and Facebook were also frequently reported as referral sources, with 8 private Facebook groups targeted and advertising expenses totaling CAD $2808.18. Additional study promotion took place on Tumblr and Discord. Advertisements on Grindr began in December 2023 but were discontinued after approximately 1 month due to high advertising costs and low recruitment yield (1/957, 0.1%).

**Table 2:** Social media impressions, views, and likes (N=7,777,218).

Social media platforms	Impressions (n=7,617,166), n (%)	Views (n=154,430), n (%)	Likes (n=5622), n (%)
Facebook	—^a^	—	43 (0.8)
Instagram	5,725,940 (75.2)	—	1447 (25.7)
Reddit (account 1)	235,772 (3.1)	—	—
Reddit (account 2: DARE^b^ study)	1,352,388 (17.8)	—	—
TikTok	51,765 (0.7)	51,765 (33.5)	2041 (36.3)
Tumblr	—	—	677 (12)
Tumblr Blaze advertisements	245,690 (3.2)	—	1021 (18.2)
X	—	102,124 (66.1)	375 (6.7)
YouTube	5611 (0.1)	541 (0.4)	18 (0.3)
Discord	—	—	—

^a^Not applicable.

^b^DARE: Detransition Analysis, Representation, and Exploration.

### Challenges and Controversies During Recruitment and Data Collection

**Figure 1:**
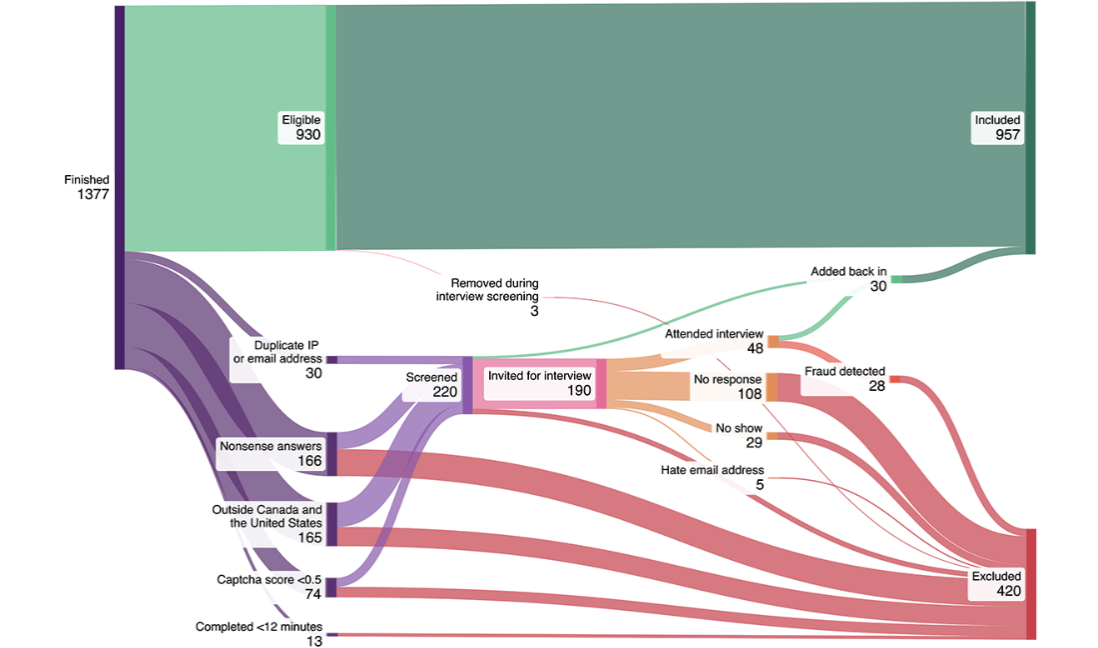
Sankey diagram of the number of survey responses removed for each criterion.

The DARE study was successful in reaching diverse and disparate web-based networks and was therefore discussed, promoted, and commented on by people with various sociodemographic backgrounds who shared divergent political perspectives as well as expectations about the study itself. These activities may have driven awareness about the study and notified more eligible people. However, it may have also biased various web-based communities’ perceptions (eg, negative or positive) of the study objectives; for example, a Tumblr post encouraged participation in the study but noted that, because the principal investigator’s past detransition research included nonbinary people, he “has an agenda” [51]. On Reddit, posts within LGBTQ2S+ and TGD subreddits introduced the DARE study as triggering feelings of “disgust” (r/LGBT) [52] or specifically encouraged TGD people who had detransitioned or retransitioned without regret to participate because “anti-trans grifters are trying to skew results of this study” (r/MtF) [53]. Five completed surveys, primarily originating from antidetransition spam email addresses, included hate speech, and these were removed by the scam and nonsense protocol (Figure 1). In private TGD or detrans-focused groups on Facebook, members occasionally questioned the study or hesitated to post recruitment flyers. A few times, Facebook group moderators removed posts about the study; however, in most cases, after the research team clarified the study’s goal of building care and support, moderators reversed the decision.

**Figure 2:**
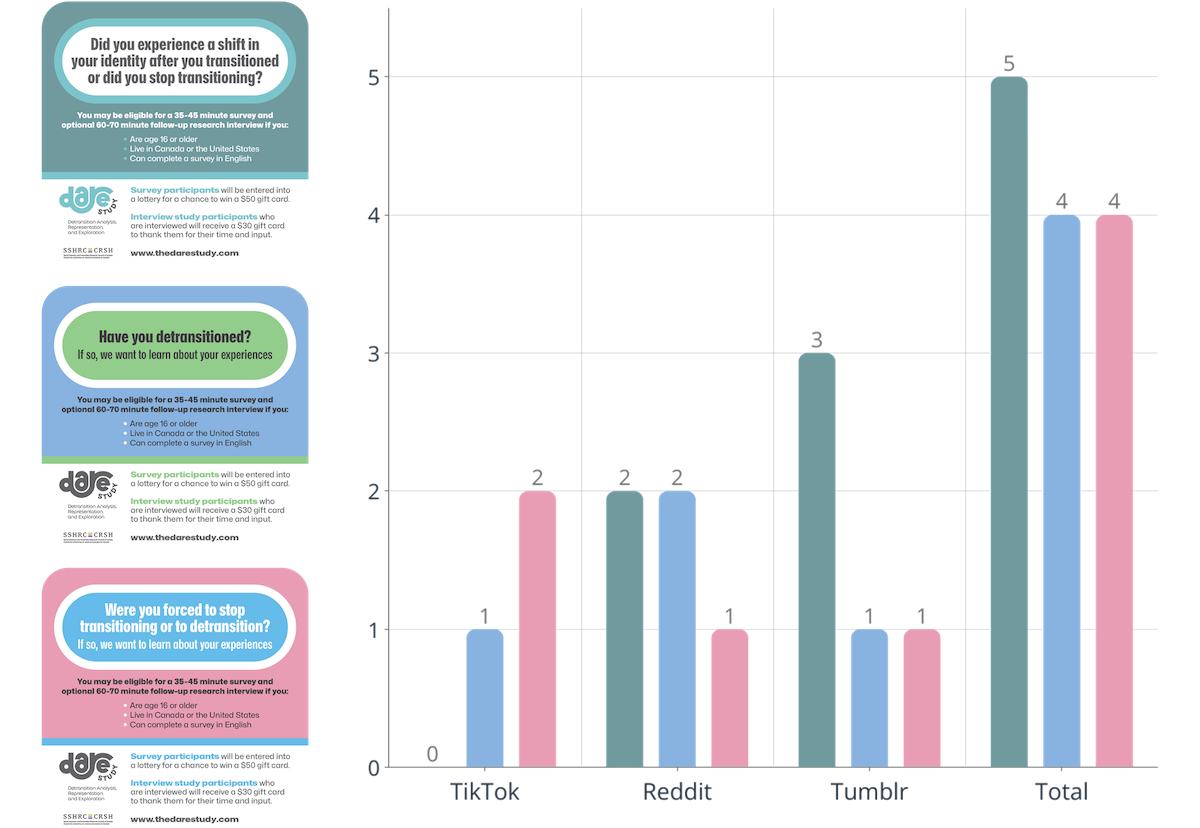
Rejected or removed paid advertisements.

Several barriers to study promotion and data collection were encountered with paid social media advertising campaigns. Some advertising campaigns were rejected outright or removed shortly after launch by Reddit, Tumblr, and X (Figure 2). After 3 weeks of carrying paid advertising for flyers 1 and 2 via the study principal investigator’s verified professional account, Reddit removed both campaigns, citing a violation of its restricted policy for promoting “health and wellness,” which required special approval [54]. The Reddit advertising division was contacted several times for support in having the advertisements approved, but these emails went unanswered. Later, using a different Reddit user account, the 3 advertisements (in all 3 languages) were successfully promoted via paid advertising campaigns for approximately 4 weeks. However, all 3 posters were eventually removed again, with Reddit citing a violation of “style” policies.

Similar issues were encountered on other platforms. Tumblr rejected several requests for paid “Blaze” advertising campaigns, while, on 2 occasions, TikTok either rejected the application for paid promotion (for flyers 2 and 3) or discontinued promoting a video after accepting payment. TikTok also temporarily suspended the principal investigator’s account after a paid promotion for the study had been purchased. Instagram and Facebook did not remove or reject any paid advertising, to our knowledge, for any of the flyers. Despite numerous emails, X never approved any advertising campaigns or responded to efforts to purchase promotions.

According to TikTok analytics, the platform’s algorithm promoted flyer 3 (“forced detransition”) to a greater degree than the other 2 flyers (Multimedia Appendix 3). This may be explained by flyer 3 receiving more “likes” and shares among users, which likely contributed to higher engagement and led the algorithm to pushing the video to a wider audience, a known feature of TikTok’s algorithm [49].

### Website Traffic, Completed Surveys, and Participant Self-Report About Study Promotion Sources

The study website had 12,272 unique visitors from December 1, 2023, to April 30, 2024, from 78 countries. To access the website, 90.1% (11,167/12,272) of the visitors used a mobile phone, 8% (982/12,272) used a desktop computer, and 1% (123/12,272) used a tablet. Of the 12,272 visitors, 11,412 (93%) were from Canada and the United States, with a majority (n=7609, 66.68%) coming from the United States. The visitors were directed from 35 different traffic sources, with the top 5 being Facebook (3,577,520/7,777,218, 46%), direct link (2,255,393/7,777,218, 29%), Reddit (1,011,038/7,777,218, 13%), Instagram (466,633/7,777,218, 6%), and X (233,317/7,777,218, 3%). These website traffic data contrasted with participants’ self-reports. For instance, survey data indicated that Tumblr (286/957, 26.7%) and Reddit (247/957, 23%) were the most frequently reported platforms where participants learned about the study. Notably, Tumblr was not a predefined survey option but was commonly selected as an “other” response. These sources were followed by Meta (198/957, 18.5%), a friend (78/957, 7%), Twitter/X (61/957, 6%), and a referral from a researcher (61/957, 6%). The remaining self-reported survey sources included TikTok, Discord, word of mouth, LGBTQ+ organizations, care providers, Grindr, and other sources (141/957, 13.2%). Despite directly emailing >1800 organizations, care providers, and former research contacts, direct email referrals did not seem to be a popular source: <7% (67/957) of the participants reported a direct email referral source (eg, from a researcher or care provider). Participants completed surveys in the following languages: English (1354/1377, 98.3%), Spanish (5/1377, 0.4%), and French (18/1377, 1%).

Of the 190 participants suspected of scam who shared their e-mail address and were invited to verify their responses via a Zoom interview, 48 (25.3%) attended. After the screenings, we excluded 28 (58%) of these 48 participants. After applying the scam screening exclusion protocol and conducting Zoom screenings, 957 (69.5%) of the 1377 completed survey responses were determined to be eligible and included in the analytic dataset. Figure 1 and Table 3 highlight this process, including the number of, and rationale for, exclusions.

**Table 3:** Total exclusions before Zoom screenings. The exclusion criteria were applied in the order listed in the table (n=448).

Exclusion criteria	Participants excluded, n (%)
Ineligible and nonsense answers	166 (37.1)
Survey completed outside Canada and the United States	165 (36.8)
reCAPTCHA^a^ score of <0.5	74 (17)
Duplicated “identity” (duplicate email or IP address)	30 (7)
Survey completion time <12 min	13 (3)

^a^CAPTCHA: Completely Automated Public Turing Test to Tell Computers and Humans Apart.

### Sociodemographics and Geographic Distribution of the Analytic Sample

After exclusions, 957 eligible participants were included in the analytic dataset. The sample was homogenous in terms of race and sex assigned at birth. A large majority identified as White (Canadian, American, or of European descent; Table 4), and a large majority were AFAB (754/953, 79.1%). Out of 957 participants, 4 (0.4%) skipped the question on sex recorded on birth certificate.

**Figure 3:**
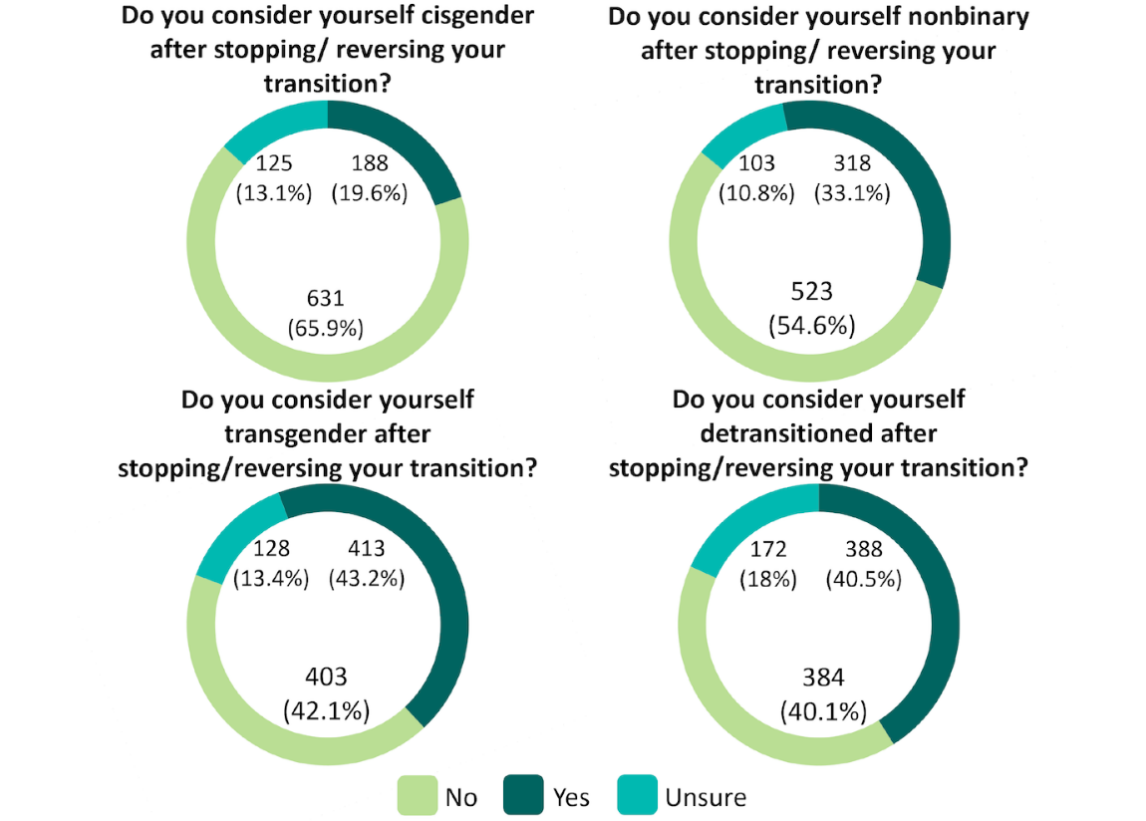
Self-identification as cisgender, nonbinary, transgender, and detransitioned. Participants could select “yes” to >1 option.

**Figure 4:**
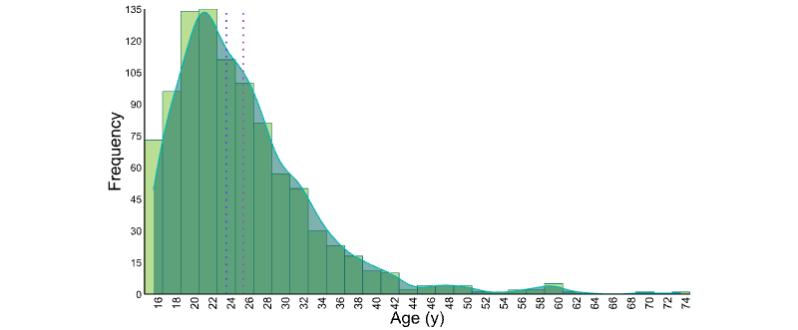
Age distribution of the analytic sample (n=957; mean 25.87, SD 7.77 y; median 24 y).

Participants were diverse in terms of age distribution and SGM identities (eg, TGD, detransitioned, cisgender, bisexual, lesbian, gay, heterosexual, and asexual; refer to Figure 3 and Table 5 for a complete breakdown of SGM status). As shown in Figure 4, the mean age of the sample was 25.87 (SD 7.77; range 16-74) years.

**Figure 5:**
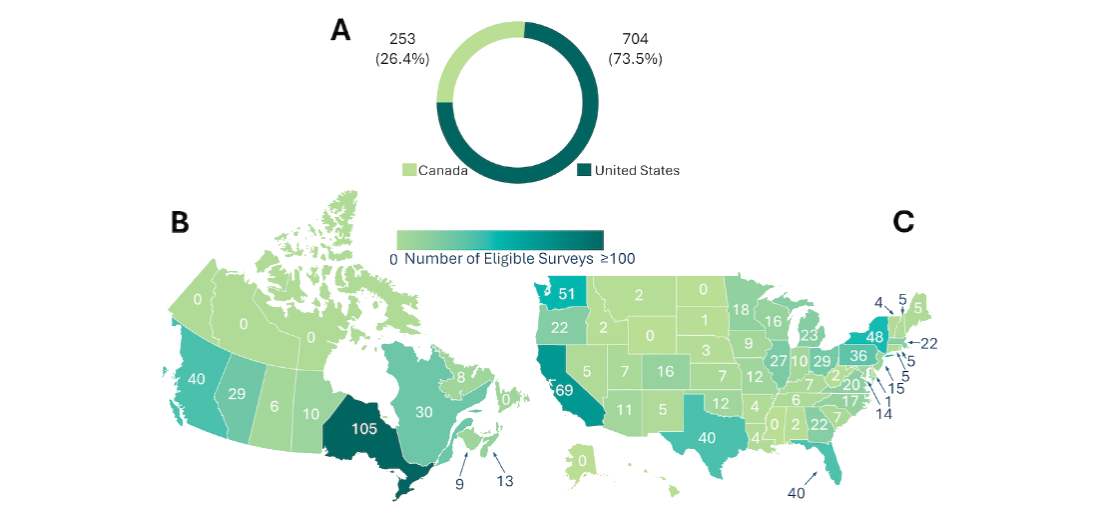
Geographic distribution of the analytic sample. (A) Pie diagram illustrating the proportions and number of responses from participants living in Canada and the United States. (B) Number of responses from each province in Canada. (C) Number of responses from each state in the United States (1 participant from Washington, DC, was included in the count for Virginia, and 1 participant was living in Puerto Rico [not included in the image]).

A majority of the participants were living in the United States (704/957, 73.6%). While the geographic distribution of the sample was fairly representative, with participants from most of the US states and Canadian provinces (Figure 5), there were a few provinces and states with no participants, such as Canada’s northern territories (Yukon, Northwest Territories, and Nunavut) and Prince Edward Island, as well as Alaska, North Dakota, Wyoming, and Mississippi in the United States.

**Table 4:** Self-identified racial and ethnic backgrounds. Of the 957 participants, 7 (0.7%) skipped this question. Participants could choose multiple options.

Racial and ethnic backgrounds	Participants (n=950), n (%)
Black African (eg, Ghana, Kenya, or Somalia)	12 (1.3)
Black Canadian or African American	33 (3.5)
Black Caribbean (eg, Jamaica or Haiti)	13 (1.4)
Central Asian	1 (0.1)
East Asian (eg, China, Japan, Korea, or Taiwan)	36 (3.7)
Indigenous (eg, First Nations, Metis, Inuit, or American Indian)	76 (7.9)
Indo-Caribbean (eg, Guyanese with origins in India)	2 (0.2)
Jewish	98 (10.2)
Latin American (eg, Argentina, Mexico, or Nicaragua)	70 (7.3)
Middle Eastern (eg, Egypt, Iran, Israel, or Saudi Arabia)	26 (2.7)
Pacific Islander or Polynesian	2 (0.2)
South Asian (eg, India, Sri Lanka, or Pakistan)	4 (0.4)
Southeast Asian (eg, Vietnam, Malaysia, or the Philippines)	14 (1.5)
Romany	1 (0.1)
White Canadian or White American	748 (78.2)
White European (eg, United Kingdom, Greece, Sweden, or Russia)	194 (20.3)

**Table 5:** Sexual orientation identity. Of the 957 participants, 2 (0.2%) skipped this question. Participants could choose multiple options.

Sexual orientation identity	Participants (n=955), n (%)
Bisexual	429 (44.8)
Queer	277 (28.9)
Lesbian or homosexual	254 (26.5)
Pansexual	114 (11.9)
Straight or heterosexual	108 (11.3)
Asexual	101 (10.6)
Gay or homosexual	94 (9.8)
Not sure or questioning	73 (7.6)
2-spirit	8 (0.9)

## Discussion

### Summary

The DARE study attracted a large and diverse US and Canadian sample of 957 LGBTQ2S+ individuals with experience of stopping, shifting, or reversing an initial gender transition. To obtain this nonprobability sample, >1800 organizations, care providers, and former research participants living in the United States or Canada were individually emailed information about the study. A major advertising and recruitment strategy involved paid study promotions (featuring 3 flyers in 3 different languages) totaling >CAD $7400 (US $5551) on popular social media platforms and social networking sites. We reached >7.7 million accounts. These efforts attracted 12,272 unique visitors to our study website, with 1377 (11.22%) completing the survey. Compared to study promotions on other platforms, those on Instagram and Facebook encountered fewer barriers to advertising and were among the top referral sources to the study website, a finding consistent with a prior SGM web-based survey [16]. In light of these results, we discuss and reflect on the Sisyphean efforts to sampling and recruitment procedures for this community-driven study, as well as the strengths and limitations of our novel approach.

### Study Promotion and Recruitment With LGBTQ2S+ and Detrans Communities

The DARE study encountered both support and challenges in study promotion and data collection, as reflected by public posts on Reddit and Tumblr as well as difficulties in placing advertisements. Given the prominence of antitransgender rhetoric and the politicization of detransition by right-wing actors [55], SGM people who have detransitioned may be reluctant to participate in studies designed to examine their experiences due to concerns about nefarious actors. This may also explain some of the discussion about the study in the r/LGBT and r/MtF subreddits, as well as several malicious antidetransition responses received. Given this, despite a large majority of our participants identifying as SGM, many LGBTQ2S+ organizations and gender clinics might have felt unable to show outward support for this research [56]. This could explain why only a small minority of participants (15/957, ~2%) reported these referral sources.

As well, some detransitioned people disconnect from LGBTQ2S+ communities or gender clinics, a phenomenon identified in previous research [36,42]. People with fluid gender identities are also more likely to avoid health care than gender-consistent TGD individuals [57]. These challenges were proactively anticipated when designing the study, which informed the decision to use social media advertising and promote multiple study flyers—particularly flyer 2 (“stopping transition or identity shift”) and flyer 3 (“forced detransition”). Although it is possible that even including the term “detransition” in our study title created apprehension, SGM researchers should note that entirely avoiding using “detransition” in favor of alternative, euphemistic terminology risks alienating some people who relate to this language [7]. Due to polarization, particularly within both TGD and detrans community networks, there can be mistrust of researchers. Language choices and advertising designs that prioritize one detransition experience over another may inherently create selection bias. Using multiple, relatively neutral flyers tailored to include disparate perspectives and experiences is one solution that seemed to attract heterogeneous identities.

### Bot, Scam, and Nonsense Detection and Exclusion Criteria: Lessons for Researchers

Potentially amplified by the US $50 or CAD $50 (US $37) gift card raffle incentive, the study attracted many scam as well as ineligible and nonsense survey responses. Given that more than a quarter of the completed surveys (420/1377, 30.5%) were deemed ineligible, our results in identifying and excluding scam and nonsense survey responses offer important lessons on internet-based sampling. Early in the data collection phase, we realized that many of the completed survey responses seemed suspicious; therefore, we created a protocol to identify and remove these responses from the dataset (refer to the Methods section). Making these adjustments required a protocol amendment and approval from the research ethics board. The scam screening process, including email communications and virtual video interviews, was both time and resource intensive. Despite these additional time and resource costs, the Zoom screening process enabled us to confirm that, of the 220 responses flagged as suspicious, 30 (13.7%) were valid and could be readded to the dataset. An additional 179 participants who agreed to be contacted for qualitative interviews for the second phase of the study were also invited to complete verification screening. These participants were not flagged as potential scam or fraud and after completing 65 preinterview Zoom screenings, only 3 scam cases were identified. Lower-resourced projects may decide to exclude all suspicious responses, given the relatively low odds of identifying eligible respondents.

### Sample Sociodemographics and Geographic Distribution

The final sample was heterogeneous in terms of LGBTQ2S+ identities, age (including adolescents, young adults, and adults aged >35 y), geographic distribution across US states and Canadian provinces, and other sociodemographic variables. Interestingly, ~11% (105/957) of the participants were living in Ontario, Canada. While Ontario is the most populous province in Canada, with 15.6 million residents [58]—of whom >39,000 are TGD individuals aged >15 years [59]—this is still disproportionate representation in comparison to many US states (eg, California).

There are a few possible explanations for this finding. First, much of the in-person study promotion and posting of physical flyers occurred at Ontario-based gender clinics and LGBTQ2S+ support spaces. In addition, a majority of the study team members (5/7, 71%) have extensive LGBTQ2S+ professional and social networks in Ontario. Another interpretation is that social media promotion was largely based out of Toronto and southern Ontario, which may have led algorithms to prioritize sharing content and advertisements locally and through our team’s existing social media networks (eg, TikTok and Instagram). A third explanation is that, in comparison to other US and Canadian regions, a Toronto-based child gender clinic was among the first in the world to begin referring children and adolescents for gender-affirming medical interventions in the early 2000s [60]. It is plausible that some of those individuals may have detransitioned as adults and participated in the study. Regions with wider access to gender-affirming health care may also see greater distribution of these experiences, whereas areas with a hostile climate toward gender diversity and poor access to gender-affirming health care may see fewer instances of detransition.

### Limitations

This study took efforts to sample a large and heterogeneous group in terms of transition-related experiences and pathways to detransition, LGBTQ2S+ identities, assigned sex and gender, race and ethnicity, age, geographic location, and other sociodemographic factors. Evenly distributing the 3 flyers via paid advertisements proved difficult, which may have created selection bias favoring some experiences over others. There were 13 total rejections and removals of the 3 flyers and disparities in views, likes, and engagement. These issues may have arisen because the study advertisements were reported by other users; for instance, TikTok is known to “shadow ban” or censor some LGBTQ2S+ content [49] and sexuality-related topics [61]. X, TikTok, and Reddit posed more substantial barriers to placing paid advertisements than Tumblr, Instagram, and Facebook. On Reddit, the study was advertised via paid or unpaid methods across 30 different subreddits, including those focused on TGD, LGBTQ2S+, detrans, and general topics. The relative success with web-based recruitment channels versus organizational and care provider referrals may have introduced a bias favoring younger participants.

Furthermore, most of the participants were White (Canadian, American, or of European descent) and AFAB (754/953, 79.1%), and were living in the United States (704/957, 73.4%). While this indicates homogeneity in these particular demographic characteristics, these demographics are also roughly consistent with the general TGD adolescent and young adult population diagnosed with gender dysphoria and who have socially or medically transitioned in North America [62,63]. Per Canadian gender clinic and community samples, approximately 80% of TGD adolescents and young adults are estimated to be transmasculine and AFAB, and a large majority are White (75%-78%) [64,65]. A representative US sample of TGD people showed that the increase in the prevalence of TGD identities observed between 2014 and 2022 was disproportionately driven by White, AFAB, young adults [66]. Still, there may be important distinctions between AMAB individuals and racialized LGBTQ2S+ people, which future analyses with this dataset will examine.

While the sample had a wide age range (16-74 y), the mean age of the sample was 25.87 (SD 7.77) years. This may be partly because a majority of the sample (811/957, 84.7%) reported learning about the study through social media and because the survey was only available on the web. In addition, those without access to a computer, smartphone, or tablet would have been unable to take the survey, which may have excluded some older individuals. Another Canadian and US study that examined TGD gender-affirming medical treatment discontinuation found that those who stopped or reversed treatment had a mean age of 22 years [65]. Overall, while many studies on detransition show samples with a majority of participants aged ≤29 years [7], greater efforts to include older adults are warranted.

To identify and remove ineligible and scam responses, we developed a protocol. We encourage other researchers to review and build from this approach because recent surges in bot and scam activity pose a threat to web-based research. We recognize that this approach may have risked excluding individuals who were indeed eligible but who misread questions or incorrectly filled out parts of the survey (eg, by accidentally indicating “yes” to a fictitious disorder question).

### Conclusions

Several new studies examining the discontinuation of gender-affirming medical treatments, detransition, and gender identity fluidity have been published recently, and this study extends this body of literature [8,67-70]. However, there is still no mutually agreed-upon or uniform conceptualization for detransition. This, together with the antitransgender politicization of these experiences and the prevalence of bot and scam responses in web-based research, presented many challenges to sampling and recruitment. Persistence in light of these challenges during the study promotion and data collection phases of our study culminated in a Sisyphean LGBTQ2S+ community–engaged project that successfully obtained a large and diverse SGM sample. As writing about detrans people now appears in major publications such as *Nature* [6], future analyses of this dataset can support the development of robust and empirically rich characterization and conceptualization of this understudied SGM experience.

## Appendix

Multimedia Appendix 1 The 21-item Likert scale (pathways to detransition).

Multimedia Appendix 2 Study flyers in English, French, and Spanish.

Multimedia Appendix 3 TikTok advertisements (breakdown).

Multimedia Appendix 4 Reddit paid and unpaid advertising (subreddits).

Multimedia Appendix 5 Facebook groups (unpaid advertising).

## Abbreviations

AFAB: assigned female at birth
AMAB: assigned male at birth
CAPTCHA: Completely Automated Public Turing Test to Tell Computers and Humans Apart
DARE: Detransition Analysis, Representation, and Exploration
LGBTQ2S+: lesbian, gay, bisexual, transgender, queer, and 2-spirit
SGM: sexual and gender minority
TGD: transgender and gender-diverse
USTS: United States Transgender Survey
