# Supplementary material for: Introducing Novel Methods to Identify Fraudulent Responses (Sampling With Sisyphus): Web-Based LGBTQ2S+ Mixed-Methods Study
Source: J Med Internet Res. 2025 Mar 17;27:e63252. doi: 10.2196/63252 (PMC11959198; doi:10.2196/63252)
Supplement: Multimedia Appendix 1 [file jmir_v27i1e63252_app1.pdf]

Appendix Materials – the DARE study

Appendix 1. 21-item Likert scale (pathways to detransition)

Section 4: Reasons for detransitioning

In the next set of questions, we will ask you questions about why you stopped transitioning, or detransitioned.

Q4.1 Please indicate to what extent the **following psychological reasons** contributed to your decision to stop or reverse your **initial transition**:[matrix question][response options:]{Not at all, A little, Somewhat, A lot}

a. My mental health did not improve while transitioning
b. My mental health was worse while transitioning
c. My healthcare provider encouraged me to address my gender dysphoria with non-medical treatment options
d. I discovered that my gender dysphoria was caused by something specific (i.e. trauma, abuse, autism)
e. My gender dysphoria resolved over time

Q4.2 Please indicate to what extent the **following physical reasons** contributed to your decision to stop or reverse your **initial transition**:[matrix question][response options:]{Not at all, A little, Somewhat, A lot}

a. I was dissatisfied by the physical results of the medical interventions
b. I was satisfied with the physical results of the medical interventions
c. My physical health was worse while transitioning
d. I had medical complications from the medical interventions

Q4.3 Please indicate to what extent the **following external reasons** contributed to your decision to stop or reverse your **initial transition**:[matrix question][response options:]{Not at all, A little, Somewhat, A lot}

a. I felt discriminated against
b. I did not have enough support in my life to continue transitioning
c. I had trouble paying for hormones or surgeries
d. Legislative bans on gender care required me to stop transitioning
e. I lost access to healthcare or insurance coverage
f. I lost my housing and there was too much instability in my life to continue transitioning
g. I continued to be perceived as transgender (i.e. I did not "pass")
h. I experienced rejection from prospective romantic/sexual partners

Q4.4 Please indicate to what extent the **following social reasons** contributed to your decision to stop or reverse your **initial transition**:[matrix question][response options:]{Not at all, A little, Somewhat, A lot}

a. My personal definition of woman or man changed and I became more comfortable with my birth sex
b. My identity changed and I no longer felt a need for medical interventions
c. I felt the changes from hormones/surgery were not enough to "pass" consistently
d. I realized that my desire to transition was erotically motivated
