# Supplementary figures and images for: Introducing Novel Methods to Identify Fraudulent Responses (Sampling With Sisyphus): Web-Based LGBTQ2S+ Mixed-Methods Study
Source: J Med Internet Res. 2025 Mar 17;27:e63252. doi: 10.2196/63252 (PMC11959198; doi:10.2196/63252)

**Appendix Materials – the DARE study**

**Appendix 2. Study flyers in English, French, and Spanish**

**
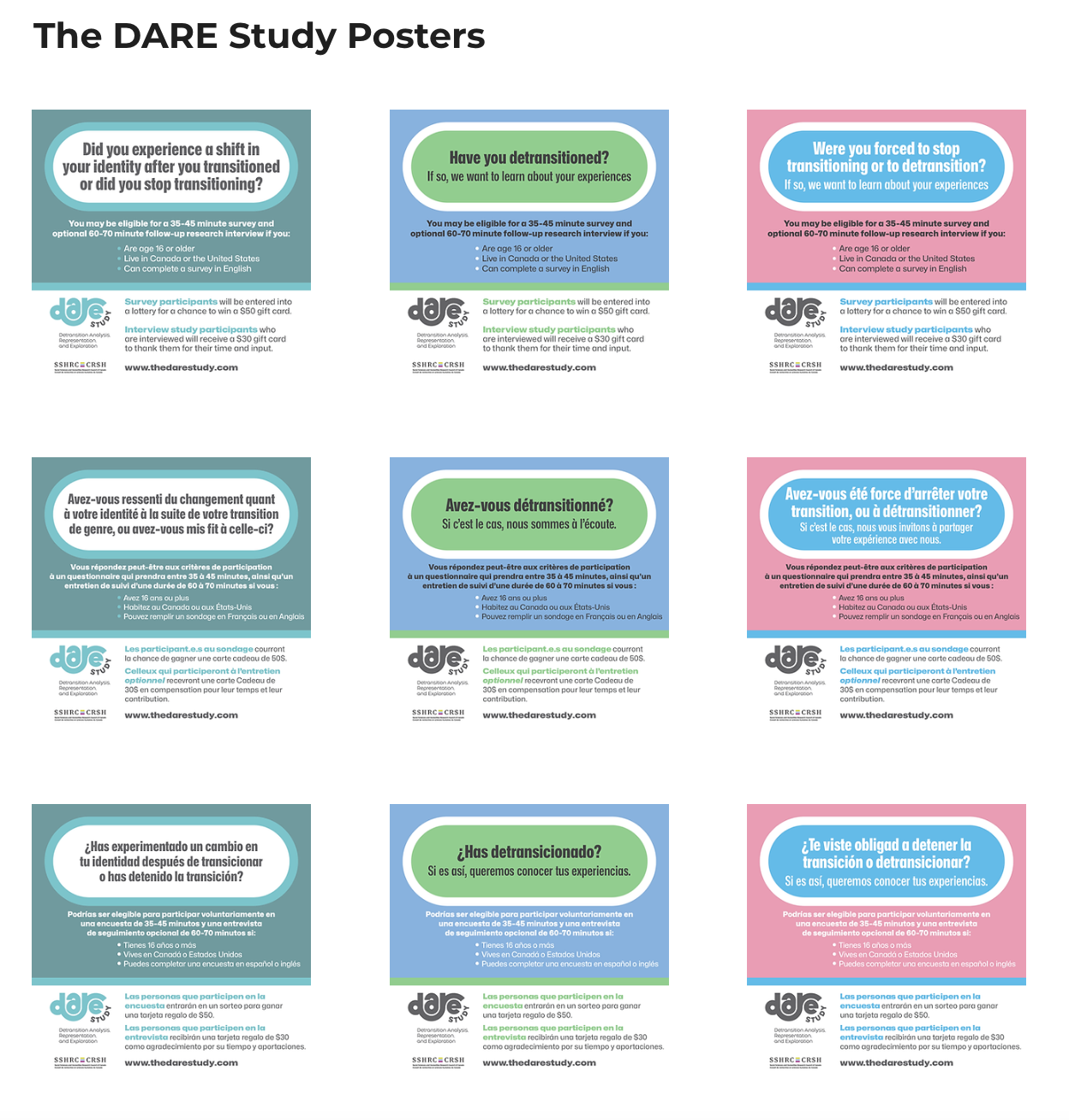
**

Supplement: Multimedia Appendix 2 [file jmir_v27i1e63252_app2.docx]
