# Supplementary material for: Introducing Novel Methods to Identify Fraudulent Responses (Sampling With Sisyphus): Web-Based LGBTQ2S+ Mixed-Methods Study
Source: J Med Internet Res. 2025 Mar 17;27:e63252. doi: 10.2196/63252 (PMC11959198; doi:10.2196/63252)
Supplement: Multimedia Appendix 3 [file jmir_v27i1e63252_app3.pdf]

Appendix Materials – the DARE study

Appendix 3. TikTok Advertisements, Breakdown

Post Date	Post/Flyer	Number of Shares by Users	Views/Impressions	Likes
22-Jan	#3 "Forced detransition"	54	86,000	997
13-Dec	General study promotion	13	26,100	747
19-Dec	#1 "Shift in identity/stop transitioning"	9	13,500	143
29-Feb	#2 "Detransition"	2	6,630	40
29-Feb	#1 "Forced detransition"	2	5,449	114
**Totals**		**80**	**137,679**	2,041
