# Supplementary material for: Introducing Novel Methods to Identify Fraudulent Responses (Sampling With Sisyphus): Web-Based LGBTQ2S+ Mixed-Methods Study
Source: J Med Internet Res. 2025 Mar 17;27:e63252. doi: 10.2196/63252 (PMC11959198; doi:10.2196/63252)
Supplement: Multimedia Appendix 4 [file jmir_v27i1e63252_app4.pdf]

Appendix Materials – the DARE study

Appendix 4. Reddit Paid and Unpaid Advertising, Subreddits

1. r/Ask_detransition
2. r/Askreddit
3. r/Askrupaulsdragrace
4. r/Askscience
5. r/Atheism
6. r/BlockedandReported
7. r/Canadasdragrace
8. r/Drag
9. r/Dragrace
10. r/Dragrace_Canada
11. r/Latina
12. r/Learnspanishinreddit
13. r/Medicine
14. r/MTFfashion
15. r/Politics
16. r/Queereye
17. r/Detrans
18. r/Actual_detrans
19. r/Honest_transgender
20. r/LGBT
21. r/MTF
22. r/MTFButch
23. r/Rupaul
24. r/Rupaulsdragrace
25. r/Science
26. r/Scienceuncensored
27. r/Spanish
28. r/Transfemininefashion
29. r/Transmascmemes
30. r/Transgender
