# Supplementary material for: Introducing Novel Methods to Identify Fraudulent Responses (Sampling With Sisyphus): Web-Based LGBTQ2S+ Mixed-Methods Study
Source: J Med Internet Res. 2025 Mar 17;27:e63252. doi: 10.2196/63252 (PMC11959198; doi:10.2196/63252)
Supplement: Multimedia Appendix 5 [file jmir_v27i1e63252_app5.pdf]

Appendix Materials – the DARE study

Appendix 5. Facebook Groups (Unpaid Advertising)

1. 2SLGBTQIA+ Educators Network
2. Detransitioners
3. EX FEMALE TO MALE DETRANSITIONERS + SUPPORTERS FOLLOWING JESUS
4. Jobs For Queers: Halifax/Dartmouth
5. Queers in health, social and community work
6. THEA (Transgender Health and Education Alliance)
7. Toronto Queer Events 🏳️‍🌈
8. Trans Family Nova Scotia
9. Trans Care BC
